# Intimate partner violence against women in the Erbil city of the Kurdistan region, Iraq

**DOI:** 10.1186/1472-6874-13-37

**Published:** 2013-10-10

**Authors:** Hazha H Al-Atrushi, Namir G Al-Tawil, Nazar P Shabila, Tariq S Al-Hadithi

**Affiliations:** 1 Ministry of Health, Erbil, Iraq; 2 Department of Community Medicine, College of Medicine, Hawler Medical University, Erbil, Iraq

## Abstract

**Background:**

Violence against women is a worldwide problem and serious human rights abuse that occurs among all social, cultural, economic and religious groups. There is a paucity of research on intimate partner violence against women in Iraq, particularly in the Kurdistan region. This study assessed the prevalence of emotional, physical and sexual intimate partner violence against women and the impact of physical violence in Erbil, the main city of the Iraqi Kurdistan region.

**Methods:**

A cross-sectional study was carried out on a convenience sample of 800 Kurdish ever married women. Women (aged 16 to 65 years) attending two public hospitals in Erbil city for reproductive health problems were included in the study. The study was conducted between 1^st^ of October 2009 and 30^th^ of March 2011. Each woman was seen only once. Intimate partner violence was assessed by administering a modified version of the World Health Organization’s domestic violence questionnaire through direct interview by a female doctor. Prevalence of intimate partner violence was assessed by timing (lifetime or past year), frequency (once, 2–5 times, > 5 times), and type (emotional, physical, and sexual violence). Descriptive statistical analysis was conducted with calculation of frequencies and percentages of women who reported different types, severities and impact of intimate partner violence.

**Results:**

The prevalence of the overall lifetime and the overall past year intimate partner violence against women was 58.6% and 45.3%, respectively. The proportions of women experienced at least one form of lifetime intimate partner violence were: 52.6% for emotional abuse; 38.9% for physical violence; and 21.1% for sexual violence, while 43.3%, 15.1%, and 12.1% of women experienced at least one form of past year emotional, physical and sexual violence, respectively. Among those with lifetime physical violence, 11.6% were subjected to more serious injuries like stab wound, broken teeth or broken bones.

**Conclusions:**

There is a high prevalence of intimate partner violence, in particular emotional abuse behavior, against the women attending hospitals in Erbil. Physical violence is also a significant problem particularly in terms of its consequences.

## Background

Violence against women is a worldwide problem and a serious human rights abuse that occurs among all social, cultural, economic, and religious groups. A wide range of negative health outcomes and even death had been recognized as consequences of violence [1]. The first ever WHO study on domestic violence recognized intimate partner violence (IPV) as the most common form of violence in women’s lives, much more than assault or rape by strangers or acquaintances. The study showed that women were at higher risk of violence at homes than in the streets and this has serious repercussions on women's health [2]. Sexually and physically abused women by intimate partners have a risk of 50% to 70% to develop gynecological, central nervous system, and stress related problems [3].

For women and girls of 16–44 years old, violence is one of the major causes of death and disability. In 1994, a study by the World Bank on ten selected risk factors facing girls and women in this age group, found rape and domestic violence more dangerous than cancer, motor vehicle accidents, war and malaria [4]. Because of its grave impact on the health and wellbeing of women and children, strategies to improve maternal and child health must include concerted and comprehensive efforts to address violence against women [5].

Intimate partner violence refers to behavior by an intimate partner that causes physical, sexual or psychological harm, including acts of physical aggression, sexual coercion, emotional abuse and controlling behaviors. This definition covers violence by both current and former spouses and other intimate partners [6]. Violence against women is a major public health and human rights concern, with IPV and sexual violence among the most pervasive forms of violence against women. Research has demonstrated a high prevalence of violence against women by intimate partners globally and the associated adverse physical and mental health outcomes, in both the short and long terms [7,8].

The WHO multi-country study on women’s health and domestic violence showed that 13% to 61% of women between 15 and 49 years old report that an intimate partner has physically abused them at least once in their lifetime and between 6% and 59% of women report forced sexual intercourse, or an attempt at it, by an intimate partner in their lifetime [2].

The issue of violence against women in Iraqi Kurdistan region has become a priority in the agenda since 2007 with advocacy of different women’s rights and civil society groups. However, there is a paucity of documented knowledge about violence against women in the region. This study is a component of a comprehensive assessment of this important issue of violence against women in Erbil, the main city of the Iraqi Kurdistan region. This component was carried out specifically to explore the prevalence of emotional, physical and sexual IPV against women and the lifetime impact of physical violence in Erbil.

## Methods

### Participants/setting

We invited a sample of 840 women (aged 16 to 65 years) living in Erbil, the main city of the Iraqi Kurdistan region, to participate in this study. The sample included women in contact with the hospitals due to reproductive health problems or their accompanied women. Of the 840 women recruited, 800 (95.2%) answered the questionnaire and 40 (4.8%) refused to answer. Characteristics of these 800 participants are shown in Table 1.

**Table 1:** Characteristics of women participated in the study

**Characteristics**		**No.**	**(%)**
Marital status			
	Married	753	(94.1)
	Widowed	35	(4.4)
	Divorced/Separated	12	(1.5)
Age group (years)			
	16-25	114	(14.2)
	25-34	276	(34.5)
	35-44	261	(32.6)
	45-54	122	(15.3)
	55-65	27	(3.4)
Age at marriage (years)			
	< 18	295	(36.9)
	≥ 18	505	(63.1)
Duration of marriage (years)			
	< 5	147	(18.4)
	5.0-15	322	(40.2)
	≥ 16	331	(41.4)
No. of children			
	0	101	(12.6)
	1-2	220	(27.5)
	≥ 3	479	(59.9)
Years of formal education			
	0*	303	(37.9)
	1- 6	262	(32.7)
	7 - 9	83	(10.4)
	10 - 12	47	(5.9)
	> 12	105	(13.1)
	*Total*	*800*	*(100.0)*

Illiterate and unschooled.*

### Measures

A modified version of the questionnaire designed for the WHO multi-country study on women's health and domestic violence against women [9] was used taking into consideration the modification of other workers [10-12] (Additional file 1). The questionnaire was translated into Kurdish language for interview purpose. The translation was validated by an independent Kurdish native fluent in English language, who translated the Kurdish version back to English to ensure accuracy. In order to protect the confidentiality of the information, names were not included in the written questionnaires.

The content validity of questionnaire was determined by a panel of five experts of different specializations. Experts’ comments were taken into consideration in modification and revision. These experts generally agreed that the questionnaire was appropriately designed and developed to measure the phenomenon underlying the study. Questions on being subjected to lifetime physical injuries from physical violence by intimate partner were included.

Prevalence of IPV was assessed by timing (lifetime or past year), frequency (once, 2–5 times, > 5 times) and type (emotional, physical, and sexual abuse). Emotional abuse was assessed with six items: insult, humiliation, threatening to divorce the respondent, threatening to marry another woman, threatening to hurt the respondent and doing things to scare the respondent on purpose. The items in the WHO multi-country study questionnaire for assessing controlling behavior were not included in this study. Physical abuse was assessed with 13 items: slapping, pushing or shaking or throwing things, twisting arm or pulling hair were classified as moderate physical abuse behaviors, whereas hitting, kicking or dragging, choking or burning and threatening with a weapon or using a weapon (gun, knife, or other object) were classified as severe. Sexual abuse was assessed with two items: using physical force to have sexual intercourse when respondent did not want to, and forcing the woman to perform sexual acts that she consider degrading or humiliating.

For each type of abuse, lifetime abuse was defined as the experience of one or more acts at any time from a current or former intimate partner. Abuse taking place within the past year was defined as acts from current partner taking place within the 12 months prior to the interview. Physical injuries were classified as minor (cuts, bruises and aches), serious (sprain, dislocation, eye or ear injuries and burns) and more serious (deep wound, broken bones and broken teeth).

### Design and procedure

This cross-sectional study was conducted in Erbil city between the 1^st^ of October 2009 and the 30^th^ of March 2011. A convenience sample of 800 Kurdish women aged 16–65 years attending two public hospitals in Erbil city for reproductive health problems and their accompanied women were included in the study. Thus, the study does not pretend to be inferred to all women in Kurdistan region.

Women who were too ill to complete the questionnaire or caring for very ill patients, women admitted to the labor room or operating theatre, women who became widow within the last year and women whom husbands were accompanying them were excluded from the study.

Widowed, separated and divorced women were excluded from the estimation of the prevalence of past year violence. The nature of the study was briefly described for each participant. All women were informed that participation in the study was voluntary and the data collected would not be used for anything except for research purpose. Women were interviewed by a female doctor in privacy and were asked not to reveal the topic of the study to their friends and family members including the husband for safety reasons. The data were collected six days per week where four to six women were interviewed each day. The first three days of the week were spent at the Maternity Teaching Hospital and the other three days at the Rizgary Teaching Hospital. Since the data were collected over more than six months and in order to avoid consecutive cases, it was made sure that each woman was interviewed only once.

A pilot study was conducted on 35 women aiming at determining the clarity and the content adequacy of the questionnaire. It also helped in estimating the time required for the data collection and identifying the limitations that might be encountered during data collection. However, this group of women was excluded from the study sample. The questionnaire was simplified and the interview time was reduced from 45–60 minutes to 20–30 minutes.

The study was approved by the Research Ethics Committee of the College of Medicine of Hawler Medical University and institutional approval was obtained from Erbil Directorate of Health, Maternity Teaching Hospital and Rizgary Teaching Hospital to carry out the study. A verbal informed consent was obtained from each participant. The guidelines of the WHO including the importance of ensuring confidentiality and privacy were followed [13].

### Statistical analysis

The Statistical Package for Social Sciences (SPSS) version 18 was used for data entry and analysis. A descriptive approach was used to calculate frequencies and percentages of women who reported different types, severities and impact of IPV.

## Results

### Socio-demographics

The mean age ± SD of study participants was 35.18 ± 9.69 years (range 17 to 65 years). Details of socio-demographic characteristics of study participants are shown in Table 1.

### Overall violence

Over 58% of study participants were subjected to any type of lifetime IPV. Emotional abuse was the most common type (52.6%) followed by physical and sexual violence (38.9% and 21.1%, respectively). Out of 753 married women (excluding widowed, separated and divorced), 45.3% experienced past year violence where 43.3%, 15.1%, and 12.1% of women were subjected to emotional, physical and sexual violence, respectively.

### Emotional abuse

Emotional abuse was the most common type of lifetime IPV (52.6%). High proportions of women were subjected to insult (40%) and intimidation or scaring (38.3%), compared to 12.6%, 12%, and 11.5% of the total sample who were subjected to threat to marry another woman, harm, and divorce by their partners during their life, respectively. One fifth of the study participants were subjected to humiliation. More than half of victims of emotional abuse have been exposed to such acts many times (> 5 times) during their lives. Emotional abuse was again the most common type of past year IPV (43.3%). Slightly less than one third of women were subjected to insult and scare in the past year (32.3% and 31.6%, respectively). Details of lifetime and past year emotional abuse by intimate partners are shown in Table 2.

**Table 2:** Prevalence of lifetime and past year emotional abuse by intimate partner against women

**Type of violence**	**Prevalence (n = 800)**		**Frequency of violence**					
			**Once**		**2 – 5 times**		**>5 times**	
	**No.**	**(%**)	**No.**	**(%)**	**No.**	**(%)**	**No.**	**(%)**
*Lifetime (overall)*	421	(52.6)						
Insult	320	(40.0)	8	(2.5)	131	(40.9)	181	(56.6)
Scaring/intimidation	306	(38.3)	11	(3.6)	126	(41.2)	169	(55.2)
Humiliation	165	(20.6)	4	(2.4)	34	(20.6)	127	(77.0)
Threatening to remarry	101	(12.6)	2	(2.0)	28	(27.7)	71	(70.3)
Threatening to harm	96	(12.0)	4	(4.2)	21	(21.8)	71	(74.0)
Threatening to divorce	92	(11.5)	6	(6.5)	19	(20.7)	67	(72.8)
*Past year (overall)*	326	(43.3)						
Insult	243	(32.3)	7	(2.9)	110	(45.3)	126	(51.8)
Scaring/intimidation	238	(31.6)	8	(3.4)	114	(47.9)	116	(48.7)
Humiliation	130	(17.3)	2	(1.5)	37	(28.5)	91	(70.0)
Threatening to remarry	66	(8.8)	5	(7.6)	16	(24.2)	45	(68.2)
Threatening to harm	61	(8.1)	5	(8.2)	13	(21.3)	43	(70.5)
Threatening to divorce	55	(7.3)	4	(7.3)	13	(23.6)	38	(69.1)

### Physical violence

Physical violence constituted 38.9% of lifetime IPV. The most frequent act of physical violence was slapping on face (35.5%) and a low proportion of the participants reported chocking or burning (3.0%). Prevalence of the past year physical IPV was 15.1%, where slapping was still regarded as the commonest type and choking or burning was considered the least common. The lifetime prevalence of moderate physical violence was greater than that of severe physical violence (21% vs. 17.9%). In contrast, the past year prevalence of severe physical violence was higher than moderate physical violence (8.9%, vs. 6.2%). Details of lifetime and past year physical IPV against women are shown in Table 3. Two hundred and eighty two women (40.3%) out of the 699 women who had children were subjected to lifetime physical IPV. Nearly half of physical attacks (49.3%) had been committed in front of women’s children.

**Table 3:** Prevalence of lifetime and past year physical violence by intimate partner against women

**Type of violence**	**Prevalence (n = 800)**		**Frequency of violence**					
			**Once**		**2 – 5 times**		**>5 times**	
	**No.**	**(%**)	**No.**	**(%)**	**No.**	**(%)**	**No.**	**(%)**
*Life time (overall)*	311	(38.9)						
*Moderate physical violence (overall)*	168	(21.0)						
Slapping on face*	284	(35.5)	70	(24.6)	109	(38.4)	105	(37.0)
Pushing/shaking/throwing something*	137	(17.1)	17	(12.4)	38	(27.7)	82	(59.9)
Twisting arm/pulling hair*	114	14.3)	8	(7.0)	35	(30.7)	71	(62.3)
*Severe physical violence (overall)*	143	(17.9)						
Hitting with fist/something†	135	(16.9)	16	(11.9)	45	(33.3)	74	(54.8)
Kicking/dragging†	103	(12.9)	9	(8.7)	31	(30.1)	63	(61.2)
Threatening/attacking with knife or gun†	27	(3.4)	2	(7.4)	5	(18.5)	20	(74.1)
Choking/burning†	24	(3.0)	8	(33.3)	1	(4.2)	15	(62.5)
*Pat year (overall)*	114	(15.1)						
*Moderate physical violence (overall)*	50	(6.2)						
Slapping on face*	105	(13.9)	21	(39.9)	49	(20.0)	35	(33.3)
Pushing/shaking/throwing something*	63	(8.4)	6	(9.5)	25	(39.7)	32	(50.8)
Twisting arm/pulling hair*	62	(8.2)	9	(14.5)	22	(35.5)	31	(50.0)
*Severe physical violence (overall)*	71	(8.9)						
Hitting with fist/something†	65	(8.6)	13	(20.0)	25	(38.5)	27	(41.5)
Kicking/dragging†	52	(6.9)	9	(17.3)	19	(36.5)	24	(46.2)
Threatening/attacking with knife or gun†	16	(2.1)	2	(12.5)	1	(6.3)	13	(81.3)
Choking/burning†	11	(1.5)	1	(9.1)	0	(0.0)	10	(90.9)

*Moderate physical act.

†Severe physical act.

### Sexual violence

Prevalence of lifetime sexual IPV against women was 21.1%. Nearly 21% of the women reported that they had been physically forced to have sexual intercourse during their life by husband and two thirds of them had experienced such act many times (> 5 times). In comparison, 10.8% of the women reported that they were forced to perform sexual acts that they considered degrading or humiliating. Prevalence of past year sexual IPV against women was 12.1%. Eighty eight women (11.7%) were continuously subjected to having sexual intercourse by physical force in past year and 63.6% of these victims had experienced such act many times (> 5 times). Details of prevalence of lifetime and past year sexual IPV against women are shown in Table 4.

**Table 4:** Prevalence of lifetime and past year sexual violence by intimate partner against women

**Type of violence**	**Prevalence (n = 800)**		**Frequency of violence**					
			**Once**		**2 – 5 times**		**>5 times**	
	**No.**	**(%**)	**No.**	**(%)**	**No.**	**(%)**	**No.**	**(%)**
*Lifetime (overall)*	169	(21.1)						
Physically forced to have sexual intercourse	166	(20.8)	9	(5.4)	45	(27.1)	112	(67.5)
Forced to perform a sexual act considered degrading or humiliating	86	(10.8)	3	(3.5)	32	(37.2)	51	(59.3)
*Past year (overall)*	91	(12.1)						
Physically forced to have sexual intercourse	88	(11.7)	4	(4.6)	28	(31.8)	56	(63.6)
Forced to perform any sexual act considered humiliating	52	(6.9)	3	(5.8)	23	(44.2)	26	(50.0)

### Consequences

Around 43% of physically abused women by intimate partners had experienced at least one type of physical injuries during their life. Nearly all of the injured women reported minor injuries (cuts, bruises or aches). More than one tenth of physically abused women experienced serious injuries like eye injuries, sprain, dislocation or burns during their life. Over 11% of physically abused women experienced more serious injuries like stab wounds, broken bones or broken teeth (Table 5).

**Table 5:** Consequences of lifetime physical violence (n = 311)*

**Type of consequences**	**No.**	**(%)**
*Physical injury (overall)*	134	(43.1)
Cuts, bruises or aches	132	(42.4)
Eye injuries, sprain, dislocation or burns	34	(10.9)
Stab wounds, broken bones or broken teeth	36	(11.6)

*Multiple injuries were possible.

## Discussion

### Overall violence

This study showed a high prevalence of lifetime IPV (58.6%) against women. This prevalence is similar to those reported in a hospital-based study in Baghdad (58%) [14], primary health care centers in Madina, Saudi Arabia (57.8%) [9], Sivas, Turkey (52%) [15], Eastern India (56%) [16] and Jahrom, Iran (64.7%) [17]. This prevalence is considerably higher than the rates reported among women attending general practice in some other countries and cities such as Japan (14.3%) [18], Norway (26.8%) [19], China (43%) [20], Esfahan, Iran (36.8%) [21] and Ireland (39%) [22]. The wide discrepancies in prevalence of violence may reflect different definitions for violence in every society, the method of screening, religious beliefs and cultural issues [23]. The prevalence of IPV in this study reflects wives being ill-treated by husbands, whereas most similar research in the WHO multi-country study and other countries reflect acts of abuse by “intimate partners”, which includes the spouse, ex-spouse, current/former boyfriends or current/former dating partner [24].

The prevalence of lifetime emotional, physical and sexual IPV was high in this study (52.6%, 38.9%, and 21.1% respectively). Another study from Erbil city that assessed domestic violence (by partners and non-partners) reported a lower prevalence of emotional, physical, and sexual violence (34.0%, 21.6%, and 12.1%, respectively) [25]. The Iraqi family health survey (IFHS) which was conducted between 2006 and 2007 showed a considerably lower prevalence of emotional and physical IPV against women in Kurdistan region (17.6% and 10.9%, respectively) [10]. In Baghdad, 57.3%, 39.8%, and 14.9% of women attending Al-Kadhimiya and Al-Kindy teaching hospitals reported emotional, physical, and sexual IPV, respectively [14], while in southern and central parts of Iraq 35.7% and 22.7% of women reported emotional and physical violence, respectively [10]. These low rates in the IFHS could be attributed to the study design of the IFHS which was not specific to the problem of violence only, but included different indicators simultaneously. In addition, the IFHS was a household survey where women expectedly cannot speak freely.

To our knowledge, this study is the first survey in Erbil city to assess the past year IPV. Prevalence of past year IPV in this study (45.3%) is similar to those reported in Baghdad (44%) [14], and in Omdurman, Sudan (41.6%) [26]. However, it is lower than that reported in Madina, Saudi Arabia (58.7%) [12] and higher than that reported in China 26% [20] and Esfahan, Iran 29.3% [21].

Despite the increasing efforts of Iraqi Kurdistan Regional Government by building up civil societies and women’s organizations and establishing new police directorate to deal with cases of violence against women and repealing some of the previous Iraqi laws, violence against women continues. According to the Directorate to Trace Violence Against Women in 2009, there were 37, 29 and 19 women’s murders in Erbil, Sulaymaniya, and Duhok governorates, respectively; while 1357, 815 and 486 women were reported victims of violence in Erbil, Sulaymaniya, and Duhok governorates, respectively [27].

### Emotional abuse

More than half of women in this study experienced at least one form of emotional abuse. The additional items of threatening to divorce and threatening to remarry represent aspects of emotional abuse that are relevant to women in Erbil but are not included in the WHO multi-country study instrument. These items were also included in IFHS [10]. Nevertheless, it is within the range of the WHO multi-country study; between 20% and 75% of women had experienced one or more of these acts, mostly within 12 months [2]. This rate is nearly similar to those reported in Jahrom, Iran (53.5%) [17], Esfahan, Iran (44.8%) [21], Sivas, Turkey (53.8%) [15], and in Eastern India (52%) [16]. However, it is higher than the rate reported in Madina, Saudi Arabia (32.8%) [12]. Much higher rates of verbal violence were reported in Jordan (73.4%) [11] and Karachi, Pakistan (97.5%) [28]. These differences in prevalence of each type of violence are expected given that the estimates tend to increase in response to broader definitions of the type of violence. Another reason is that the cultural background of male dominated societies further raises the prevalence of violence against women.

### Physical violence

The prevalence of lifetime physical IPV in this study (35.5%) is within the range of prevalence in some areas investigated in the WHO multi-country study across different cultures and socio-economic settings (30%-40%) such as Namibia, Bangladesh, New Zealand, Thailand, Tanzania, Brazil, and Australia [23,29]. It is slightly lower than those reported among women attending general health practice in Ireland 39% [22], Sanandaj city, Iran 38% [30] and in Sivas, Turkey 38.3% [15]. However, it is slightly higher than the rates reported among a sample of reproductive health clinic attendees in Jordan (31.2%) [11], at a national community based study in Egypt (34%) [31], among a sample of pregnant women in Jahrom, Iran (34.7%) [17] and in Esfahan, Iran (31.9%) [21]. Much higher rates of lifetime physical violence by partners were reported in rural Bangladesh (67%) [32], in low socioeconomic communities in Karachi, Pakistan (80%) [28] and Ethiopia (49%) [29]. However, much lower figures were reported in Eastern India (16%) [16], Cambodia (18%) and Vietnam (25%) [29].

The prevalence of past year physical violence (15.1%) is similar to those reported among women attending health care facilities in Sanadaj and Babol of Iran (15%) [30,33], Dar es Salaam, Tanzania (15%) and Matlab, Bangladesh (16%) [29]. A national community based study in Egypt revealed a rate of 13% [29]. A higher proportion was reported in Israel (32% among Arab population and 52% in Gaza Strip) [29], Syria (25-26%) [34,35] and rural Bangladesh (34.6%) [32].

The rate of past year severe physical abuse was 8.9%. A higher proportion was reported by women attending primary care facilities in Madina, Saudia Arabia, 16.3% [12], Egypt 26.6% [36] and in rural Bangladesh 17.3% [32]. However, this rate is higher than that reported in Japan city 0.7%, Serbia and Montenegro 1.6%, Thailand 5.1%, and Brazil (3.3% and 7.5%) [2].

The lifetime prevalence of severe physical violence of 17.9% in this study is within the range reported for most of the countries in the WHO multi-country study (13-26%) [2]. However, only three countries (Bangladesh, Japan and Serbia and Montenegro) showed similar findings to this study in terms of having a greater proportion of women experienced only moderate violence than had experienced severe violence.

### Sexual violence

The prevalence of lifetime sexual IPV (21.1%) is higher than in Baghdad 14.6% [14], but it is within the range of WHO multi-county study, where most areas fall between 10% and 50% [2]. Such similarity to the WHO multi-country study sustains the results of this study as it is based on the same questionnaire and definitions of sexual violence.

Prevalence of past year sexual abuse in this study was around 12.1%. Similar findings were reported in China (12%) [20], Samoa (11.2%) and Tanzania (12.8%) [2]. Higher rates were reported in Bangladesh (20.2% and 17.1%), Thailand (15.6%) and Tanzania 18.3% [2]. Much higher rates were reported in Ethiopia (44.4%) [2] and Babol of Iran (42.2%) [33]. These variations in the prevalence of violence between international studies and this study can be explained by differences in the study setting, study design, and characteristics of the population.

### Consequences

Among physically abused women by husbands; 43.1% had been ever injured; 10.9% reported eye injuries, sprains, dislocations or burns; and 11.6% reported more serious injuries as deep wound, broken bones or broken teeth. This finding is comparable with those of population-based studies which revealed that 40– 72% of all women who have been physically abused by a partner are injured at some point in their life [37]. In Baghdad, domestic violence by husbands was associated with minor injuries in 44.7% of cases and permanent deformity in 10.9% of cases [14]. A study in the United States of America revealed that 256 intentionally injured women had a total of 434 contusions and abrasions, 89 lacerations and 41 fractures and dislocations [38]. A WHO multi-country study reported that in Thailand over 20% of ever-injured women reported that they had been injured more than five times and at least 20% of ever-injured women in Namibia, Peru, Samoa, Thailand, and Tanzania reported injuries to the eyes and ears [23]. The self-completion module of the 2001 British Crime Survey showed that injuries were often sustained as a result of domestic violence, especially among women. During the worst incidents of domestic violence of women, 46% sustained minor physical injuries, 20% moderate physical injuries and 6% severe physical injuries [39]. In Ireland, a study found that 46% of women attending the general practice who experienced violent behavior had been injured [22].

The proportion of emotional distress and suicidal thought behaviors and attempts among physically abused women this study was 47.6% and 32.8%, respectively. This rate agrees with the finding of the self-completion module of the 2001 British Crime Survey which reported that 31% of women who were subjected to domestic violence developed mental or emotional problems [39]. Higher rates of emotional distress and suicide were reported in Baghdad (71.9%) [14]. In Nicaragua, 70% of cases of emotional distress were attributed to IPV [40]. Studies in New Zeland and Spain and the WHO multi-country study, however, revealed negative association between partners’ violence and mental health [41].

As the children who live in violent homes are at increased risk of child abuse [42], increased attention is now being focused on the children who witness domestic violence. Research estimates that approximately 3.3 to 10 million children witness the abuse of parent or adult caregiver each year [43]. The American Academy of Pediatrics and its members declared that the abuse of women is a pediatric issue. Identifying and intervening on behalf of battered women may be one of the most effective means of preventing child abuse [44]. Nearly half of physical violence acts in this study occurred in front of women’s children. These children may perceive such behavior as usual and acceptable, thus ensuring that the violence will continue. A study conducted at the scenes of police calls for domestic assault in Memphis, Tenn in 1995 found a much higher rate (85%) of children between the ages of 2 and 17 years old directly witnessed a physical assault [45]. A study from Nicaragua also showed a high rate (49%) of children who have witnessed a physical assault [40]. A review of more than 36 studies indicated that approximately 30-60% of children whose mothers are being abused are themselves likely to be abused [46]. More research is needed to develop appropriate screening tools and intervention strategies for children who are at risk.

### Limitations

This study has a number of limitations. The findings cannot be inferred to all women in Iraqi Kurdistan region as the study included a hospital-based convenience sample of participants from Erbil city only. The study is subjected to selection bias due to the different inclusion and exclusion criteria. For example the women whom husbands accompanied them were excluded from the study to allow women feel free to talk about such sensitive issue. These women might be significantly different and possibly under more controlling behaviour than those whose husbands did not accompany them. Controlling behaviour and other forms of domestic violence were not included in the study. Recall bias may significantly underestimate the prevalence of violence particularly for lifetime violence.

## Conclusions

This study showed that more than half of women were victims of emotional, physical, and or sexual violence at least once by their husbands, and 45.3% gave history of persistent abuse. The most common type of violence was emotional abuse. A considerable proportion of physically abused women reported injuries from such violence. Minor injuries reported in nearly all injured women. Further research to explore the determinants of IPV against women in an in-depth manner is needed.

## Abbreviations

IPV: Intimate partner violence; IFHS: Iraqi family health survey.

## Competing interests

The authors declare that they have no competing interests.

## Authors’ contributions

HHAA and NGAT conceptualized the study. HHAA, NGAT and TSAH designed the study. HHAA and NGAT collected the data. HHAA, NGAT and NPS carried out data analysis and interpretation. HHAA, NGAT and NPS prepared the manuscript. TSAH extensively reviewed and edited the manuscript. All authors read and approved the final manuscript.

## Pre-publication history

The pre-publication history for this paper can be accessed here:

http://www.biomedcentral.com/1472-6874/13/37/prepub

## Supplementary Material

Additional file 1 Questionnaire used to assess the prevalence of intimate partner violence against women in Erbil, Iraqi Kurdistan region. Click here for file
