# Supplementary material for: Intimate partner violence against women in the Erbil city of the Kurdistan region, Iraq
Source: BMC Womens Health. 2013 Oct 10;13:37. doi: 10.1186/1472-6874-13-37 (PMC3852841; doi:10.1186/1472-6874-13-37)
Supplement: Additional file 1 — Questionnaire used to assess the prevalence of intimate partner violence against women in Erbil, Iraqi Kurdistan region. [file 1472-6874-13-37-S1.pdf]

Additional file 1 - Questionnaire used to assess the prevalence of intimate partner violence against women in Erbil, Iraqi Kurdistan region

Code number: ☐

1. Marital status: **1**. Married **2.** Widowed **3.** Divorced **4.** Separated ☐

2. Age (years) : ☐

3. Age at marriage (years): ☐

4. Duration of marriage (years): ☐

5. Years of formal education : ☐

6. Do you have children? **1-**Yes **0-**No ☐

If yes: How many? ☐ Number of boys: ☐ Number of girls: ☐

7. (Does/Did) your husband **ever** say any of the following things to you?

If yes: How often? 1. Once 2. Few times (2-5 times) 3. Many times (> 5 times)

Did this happen during the **last 12 months**? If yes, how often? (If respondent not widowed, separated nor divorced). 1. Once 2. Few times (2-5 times) 3. Many times (> 5 times)

A) Insult you? Ever ☐ If yes, How often? ☐

Last 12 month ☐ If yes, How often? ☐

B) Say or do something to humiliate you? Ever ☐ If yes, How often? ☐

Last 12 month ☐ If yes, How often? ☐

C) Threaten to hurt you? Ever ☐ If yes, How often? ☐

Last 12 month ☐ If yes, How often? ☐

D) Threaten to divorce you? Ever ☐ If yes, How often? ☐

Last 12 month ☐ If yes, How often? ☐

E) Threaten to remarry? Ever ☐ If yes, How often? ☐

Last 12 month ☐ If yes, How often? ☐

F) Did or do things to scare or intimidate you on purpose (e.g. by the way he looked at you, by yelling and smashing things? Ever ☐ If yes, How often? ☐

Last 12 month ☐ If yes, How often? ☐

8. (Does/Did) your husband ever do any of the following things to you?

A) Slap you**?** Ever ☐ If yes, How often? ☐

Last 12 month ☐ If yes, How often? ☐

B) Push you, shake you, or through something at you? Ever ☐ If yes, How often? ☐

Last 12 month ☐ If yes, How often? ☐

C) Twist your arm or pull your hair? Ever ☐ If yes, How often? ☐

Last 12 month ☐ If yes, How often? ☐

D) Hit you with his fist or with something that could hurt you? Ever ☐ If yes, How often? ☐

Last 12 month ☐ If yes, How often? ☐

E) Kick you, drag you? Ever ☐ If yes, How often? ☐

Last 12 month ☐ If yes, How often? ☐

F) Try to choke you or burn you on purpose? Ever ☐ If yes, How often? ☐

Last 12 month ☐ If yes, How often? ☐

G) Threaten or attack you with a knife, gun, or any other weapon?

Ever ☐ If yes, How often? ☐

Last 12 month ☐ If yes, How often? ☐

H) Physically force you to have sexual intercourse with him even when you did not want to?

Ever ☐ If yes, How often? ☐

Last 12 month ☐ If yes, How often? ☐

I) Force you to perform any sexual acts you did not want to? Ever ☐ If yes, How often? ☐

Last 12 month ☐ If yes, How often? ☐

9. Did the following ever happen as a result of what your husband did to you?

A) You had cuts, bruises or aches? **1-**Yes **0-**No ☐

B) You had eye injuries, sprains, dislocations, or burns? **1-**Yes **0-**No ☐

C) You had deep wounds, broken bones, broken teeth or any other serious injuries?

1-Yes **0-**No ☐

10. Were your children present or did they overhear you being beaten? **1-**Yes **0-**No ☐
